# Rural-urban and racial-ethnic differences in awareness of direct-to-consumer genetic testing

**DOI:** 10.1186/s12889-018-5190-6

**Published:** 2018-02-23

**Authors:** Ramzi G. Salloum, Thomas J. George, Natalie Silver, Merry-Jennifer Markham, Jaclyn M. Hall, Yi Guo, Jiang Bian, Elizabeth A. Shenkman

**Affiliations:** 1 Department of Health Outcomes and Biomedical Informatics, College of Medicine, University of Florida, 2004 Mowry Road, PO Box 100177, Gainesville, FL 32610 USA; 2 Department of Medicine Division of Hematology/Oncology, University of Florida, Gainesville, USA; 3 Department of Otolaryngology, University of Florida, Gainesville, USA

**Keywords:** Genetic testing, Health disparities, Rural health

## Abstract

**Background:**

Access to direct-to-consumer genetic testing services has increased in recent years. However, disparities in knowledge and awareness of these services are not well documented. We examined awareness of genetic testing services by rural/urban and racial/ethnic status.

**Methods:**

Analyses were conducted using pooled cross-sectional data from 4 waves (2011–2014) of the Health Information National Trends Survey (HINTS). Descriptive statistics compared sample characteristics and information sources by rural/urban residence. Logistic regression was used to examine the relationship between geography, racial/ethnic status, and awareness of genetic testing, controlling for sociodemographic characteristics.

**Results:**

Of 13,749 respondents, 16.7% resided in rural areas, 13.8% were Hispanic, and 10.1% were non-Hispanic black. Rural residents were less likely than urban residents to report awareness of genetic testing (OR = 0.74, 95% CI = 0.63–0.87). Compared with non-Hispanic whites, racial/ethnic minorities were less likely to be aware of genetic testing: Hispanic (OR = 0.68, 95% CI = 0.56–0.82); and non-Hispanic black (OR = 0.74, 95% CI = 0.61–0.90).

**Conclusions:**

Rural-urban and racial-ethnic differences exist in awareness of direct-to-consumer genetic testing. These differences may translate into disparities in the uptake of genetic testing, health behavior change, and disease prevention through precision and personalized medicine.

## Background

A major promise of genomic research is the transformation of healthcare practice and population health through earlier diagnosis, more effective prevention and treatment of disease, and avoidance of treatment side effects [1]. Precision medicine is an emerging approach to detecting, treating, and managing disease that is based on individual variation in genetic, environmental, and lifestyle factors. Within the context of precision medicine, genetic testing is increasingly used to predict risk for common diseases and traits among individuals with a family or personal history of disease. Although identifying individuals with high-penetrance genes is a promising population health application of genomic and gene panel sequencing [2], the rapid growth in direct-to-consumer (DTC) genetic tests has led to important concerns related to knowledge gaps in their clinical validity and utility, the potential for consumer misinterpretation of results because of poor health literacy, language barriers, and the possibility of widening health-related disparities [3, 4].

Underserved groups have poorer access to genetic testing services within the general population [5]. Genetic testing is highly technical and typically requires multiple office visits and specialty referrals. Receipt of genetic testing and needed referrals, including counseling, is particularly vulnerable to access-related barriers and financial constraints such as lack of insurance and underinsurance [5].

In accordance with the evidence on access-related barriers to genetic testing services more broadly, findings from the Health Information National Trends Survey (HINTS) reveal that awareness of DTC genetic testing services, specifically, is lower among vulnerable populations, including racial and ethnic minorities and lower income individuals [6]. However, differences in awareness of these tests by geography have not been previously examined for the U.S. population. Therefore, the primary purpose of this study was to examine the overall awareness of genetic testing services by rural residents of the US compared with urban residents, and stratified across racial and ethnic groups, using multiple years of HINTS.

## Methods

Data were pooled from 4 waves (2011–2014) of the HINTS, a publically available, population-based survey of non-institutionalized, US civilians who are ≥18 years [7]. HINTS is a National Cancer Institute program to study the population-level use of cancer-related information, employing a complex probability sampling design, with details published elsewhere [7, 8]. This study was based on publically available de-identified data and was not subject to institutional board review.

The main outcome of interest, awareness of DTC genetic testing services, was derived from the following question: “Genetic tests that analyze your DNA, diet and lifestyle for potential health risks are currently being marketed by companies directly to consumers. Have you heard or read about these genetic tests?” The source of genetic testing information was a secondary outcome observed in the 2013 wave *only* as it was unavailable in other years. Participants reported their information source by answering “from which of the following sources did [they] read or hear anything about genetic tests?” with the option to choose one or more information sources (i.e., newspaper, magazine, radio, health professional, family member, social media, television, and/or the Internet).

The main predictor was rural-urban residence, defined using the US Department of Agriculture’s 2003 Rural-Urban Continuum (RUC) Code, with 10 categories based on county of residence in a metro or non-metro county and the population of the county. Codes 1–3 were designated as urban, representing commuting patterns to metro counties with populations of 250,000 or greater and codes 4–9 as rural, representing non-metro counties with populations ranging from 2500 to 20,000 [9]. Demographic variables included gender, age, race/ethnicity (non-Hispanic white, Hispanic, non-Hispanic black, and non-Hispanic other), education (less than high school, high school, technical/vocational or some college, and college graduate or post-graduate), and annual income (<$20,000, $20,000 to <$35,000, $35,000 to <$50,000, $50,000 to <$75,000, and $75,000+).

Data were analyzed with Stata 14 using jackknife replicate weights to estimate accurate variance estimates for statistical modelling. Descriptive statistics and bivariate analyses (using chi-square tests) were conducted to compare sample characteristics and information sources by rural/urban residence. A multivariate regression model of rural/urban residence as a predictor for awareness of genetic testing controlled for age, sex, race/ethnicity, education, income level, and survey year. We tested alternative models that controlled for family history of cancer and interaction terms between rural/urban residence and other respondent characteristics. Predicted marginals were derived for racial/ethnic minority group membership stratified by rural/urban residence.

## Results

Of 13,749 total respondents across 4 years, 2078 (16.7% weighted) resided in rural areas, 1958 (13.8%) were Hispanic, 1942 (10.1%) were non-Hispanic black, and 903 (6.7%) were non-Hispanic of other races. Significant differences were observed across sample characteristics by geography (Table 1). Compared with urban residents, rural residents had a higher percentage of individuals ≥50 years (49.4% vs. 40.9%, non-Hispanic whites (78.0% vs. 60.0%), with no more than a high school education (42.3% vs. 30.6%), and annual income of <$50,000 (60.2% vs. 47.4%).

**Table 1:** Sample characteristics by urban-rural classification: National Health Information Trends Survey (HINTS), 2011–2014

	Geographic location, n (%)			
	Urban	Rural	Chi-square	*P*-value
Sample size	11,671	2078		
Gender			0.815	0.367
Male	4584 (48.8)	838 (46.9)		
Female	7087 (51.2)	1240 (53.1)		
Age, years			67.833	<.001
18–34	1768 (30.8)	216 (24.8)		
35–49	2797 (28.3)	406 (25.8)		
50–64	3940 (24.6)	785 (28.3)		
65–74	1851 (8.9)	386 (11.3)		
75+	1315 (7.4)	285 (9.8)		
Race/ethnicity			306.289	<.001
NH white	6375 (60.0)	1486 (78.0)		
Hispanic	1849 (15.8)	109 (4.0)		
NH black	1745 (11.0)	197 (6.0)		
NH other	824 (7.2)	79 (3.9)		
Missing	878 (6.1)	207 (8.0)		
Education			152.018	<.001
Less than high school	1005 (11.0)	254 (13.2)		
High school graduate	2239 (19.6)	571 (29.3)		
Some college	3506 (32.3)	618 (35.4)		
College graduate	4811 (36.4)	607 (20.8)		
Missing	110 (0.7)	28 (1.1)		
Household income			92.840	<.001
<$20,000	2583 (20.0)	560 (25.4)		
$20,000 to <$35,000	1701 (13.9)	374 (17.8)		
$35,000 to <$50,000	1640 (13.5)	329 (17.0)		
$50,000 to <$75,000	1922 (17.0)	342 (16.9)		
$75,000+	3577 (33.9)	448 (21.5)		
Missing	248 (1.8)	25 (1.3)		
HINTS administration			7.769	0.051
2011 (HINTS 4 Cycle 1)	3177 (25.1)	620 (24.1)		
2012 (HINTS 4 Cycle 2)	2948 (25.4)	515 (24.0)		
2013 (HINTS 4 Cycle 3)	2571 (24.4)	458 (27.2)		
2014 (HINTS 4 Cycle 4)	2975 (25.1)	485 (24.7)		

Column percentages are weighted to reflect the US population per the 2010 US Census

*NH*: non-Hispanic

Rural residents were less likely (OR = 0.74, 95% CI = 0.63–0.87) than urban residents to report awareness of DTC genetic testing (Table 2). However, among those aware of genetic testing, rural and urban residents did not significantly differ in the sources from which they learned about testing (Table 3). Overall, the most commonly reported information source was television (51.2%), followed by the Internet (50.5%); and the least commonly reported information source was health professionals (16.1%). Compared with non-Hispanic whites, racial/ethnic minorities were also less likely to be aware of genetic testing (results not shown): Hispanic (OR = 0.68, 95% CI = 0.56–0.82); non-Hispanic black (OR = 0.74, 95% CI = 0.61–0.90); and non-Hispanic other (OR = 0.71, 95% CI = 0.55–0.92). Family history of cancer and interaction terms between rural/urban residence and other participant characteristics were not significant predictors of awareness of genetic testing.

**Table 2:** Weighted multivariate logistic regression model of predictors of awareness of direct-to-consumer genetic testing services across 4 waves of the Health Information National Trends Survey (HINTS) from 2011 to 2014

	Predictors of awareness of DTC genetic testing			
	Odds Ratio (95% CI)	Beta coefficient	SE Beta	*P value*
Geographic location				
*Urban*	*Reference*			
*Rural*	0.74 (0.63–0.87)	−0.30	0.08	< .001
Gender				
*Male*	*Reference*			
*Female*	1.06 (0.94–1.19)	0.06	0.06	0.329
Age, years				
*18–34*	*Reference*			
*35–49*	0.88 (0.73–1.05)	−0.13	0.09	0.163
*50–64*	1.14 (0.96–1.35)	0.13	0.09	0.138
*65–74*	1.01 (0.84–1.23)	0.01	0.10	0.882
*≥75*	0.67 (0.54–0.83)	−0.39	0.11	< .001
Race/ethnicity				
*NH white*	*Reference*			
*Hispanic*	0.68 (0.56–0.82)	−0.39	0.09	< .001
*NH black*	0.74 (0.61–0.90)	−0.31	0.10	0.002
*NH other*	0.71 (0.55–0.92)	−0.34	0.13	0.009
*Missing*	0.62 (0.49–0.78)	−0.48	0.12	< .001
Education				
*Less than high school*	*Reference*			
*High school graduate*	1.07 (0.83–1.37)	0.06	0.13	0.611
*Some college*	1.61 (1.27–2.05)	0.48	0.12	< .001
*College graduate*	2.43 (1.90–3.10)	0.89	0.12	< .001
*Missing*	1.61 (0.92–2.82)	0.48	0.28	0.093
Household income				
* <$20,000*	*Reference*			
*$20,000 to < $35,000*	1.10 (0.88–1.36)	0.09	0.11	0.407
*$35,000 to < $50,000*	1.12 (0.89–1.40)	0.11	0.12	0.324
*$50,000 to < $75,000*	1.31 (1.05–1.63)	0.27	0.11	0.017
*$75,000+*	1.65 (1.34–2.03)	0.50	0.11	< .001
*Missing*	1.31 (0.86–2.01)	0.27	0.22	0.204
HINTS administration				
*2011 (HINTS 4 Cycle 1)*	*Reference*			
*2012 (HINTS 4 Cycle 2)*	1.72 (1.47–2.02)	0.54	0.08	< .001
*2013 (HINTS 4 Cycle 3)*	0.96 (0.81–1.13)	−0.04	0.09	0.622
*2014 (HINTS 4 Cycle 4)*	0.97 (0.82–1.14)	−0.03	0.08	0.716

*DTC* direct-to-consumer, *SE* standard error, *NH* non-Hispanic

**Table 3:** Information sources among those aware of genetic testing services, by geographic location: National Health Information Trends Survey (HINTS), 2013

		Geographic location		
	Overall	Urban	Rural	*P*-value
Sample size (n)	1078	938	140	
Information sources (%)				
Television	51.2	51.1	52.1	0.597
Internet	50.5	51.8	42.5	0.344
Newspaper	28.3	29.1	23.1	0.364
Magazine	25.1	25.6	21.8	0.720
Radio	17.9	18.5	14.0	0.230
Family member	16.6	17.6	9.8	0.310
Social media	16.2	17.0	11.2	0.694
Health professional	16.1	15.3	21.2	0.784

Column percentages are weighted to reflect the US population per the 2010 US Census

**Fig. 1:**
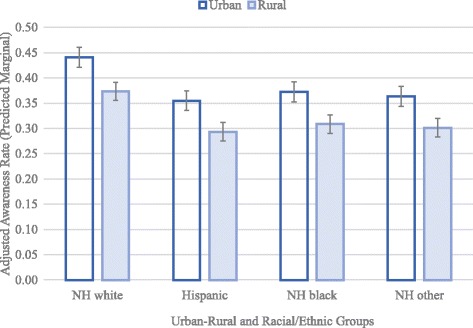
Adjusted rates (predicted marginals) for awareness of genetic testing by urban-rural and racial/ethnic categorization across 4 waves of the Health Information National Trends Survey (HINTS) from 2011 to 2014, controlling for age, sex, education, income level, and survey year

Further, predicted marginals revealed rural-urban differences across all racial/ethnic groups (Fig. 1). Predicted awareness of DTC genetic testing services was 34.4% among rural vs. 45.4% among urban non-Hispanic whites; 26.7% among rural vs. 36.7% among urban Hispanics, 28.3% among rural vs. 36.7% among urban non-Hispanic blacks; and 27.2% among rural vs. 37.3% among urban non-Hispanics of other races.

## Discussion

To our knowledge, this report is the first to examine rural-urban differences in awareness of DTC genetic testing using a nationally representative sample of US adults. After controlling for demographic characteristics and confounders, awareness of DTC genetic testing was lower among rural residents and racial/ethnic minorities compared with urban residents and non-Hispanic whites, respectively. Furthermore, the prevalence of awareness of genetic testing services among rural non-Hispanic whites was comparable to awareness among urban minorities.

Among those who were aware of DTC genetic testing, we did not observe significant differences by rural vs. urban residence in the sources of information among participants who were aware of these tests. However, this measure was only available in one year of HINTS and the lack of significant differences may be due to insufficient power. Although not statistically significant, the proportion of rural residents reporting the Internet as an information source for genetic testing was almost ten percentage points lower than among urban residents. Given that the Internet is a major information source for genetic testing, differences in Internet use or access (e.g., IT deserts) maybe contributing to geographic disparities in access to health information [10].

Meanwhile, health professionals were the least commonly reported information source, suggesting that there is an opportunity to improve delivery of or education regarding these services using interventions focused on health education and personalized medicine. However, it is worth noting that the question of interest specified DTC genetic testing, an approach to marketing genetic tests to consumers without the involvement of health professionals. DTC tests can have significant risks and limitations and health professionals may be well aware and rightfully do not discuss the tests with their patients. However, it is important for health professionals to be versed in these tests so that they properly advise patients and assist them with interpreting and acting upon the results.

Accordingly, DTC genetic testing in itself is not a substitute for a precision medicine approach to risk assessment, prevention, diagnosis, or disease management. Whereas genetic testing within the context of precision medicine promotes individualized care, a population-based approach to its implementation will ensure that its reach is extended to underserved populations and reduce health disparities. More inclusive approaches for measuring disease and susceptibility could allow for better assessment of population health and development of policies and targeted programs for preventing disease [11]. Genetic testing in the clinical setting has typically required multiple office visits; however, there have been recent developments related to the provision of genetic counseling by telephone. Two prior trials have demonstrated the equivalence of the provision of genetic counseling by telephone [12, 13], a modality that is likely to improve access for rural populations.

This study has several strengths including the use of a large, multiyear, nationally representative sample of US adults to examine differences in awareness of genetic testing by geographic location. To our knowledge, this is the first study to report on awareness of genetic testing services across racial/ethnic groups stratified by rural-urban residence. As with any cross-sectional survey design, the limitations of this study include low response rates and inability to infer causation. In addition, we were unable to assess rural-urban differences in actual use of genetic testing because this follow-up measure was only available in 1 of the 4 waves of HINTS. Finally, because we used HINTS data dating back to 2011, we were unable to analyze all waves using the updated RUC codes from 2013, and therefore we applied the 2003 definitions for consistency. Finally, we were unable to control for health literacy, a potentially confounding variable that was unavailable in HINTS.

## Conclusions

In conclusion, we found significant differences in awareness of DTC genetic testing by rural-urban residence and these differences persisted across all racial and ethnic groups. Whereas differences in awareness of DTC tests may transcend to awareness of genetic testing more broadly, a distinction should be made between the level of awareness, the appropriate use of recommended genetic services and the currently limited evidence regarding health benefits following DTC testing. Accordingly, there are clear differences between the utility of information obtained from DTC genetic tests and the need for greater awareness of appropriate use of genetic services among high risk populations within the context of precision medicine. Nonetheless, it is likely that precision medicine programs or protocols that target rural or urban residence may suffer from limitations related to this knowledge gap. Future research should focus on interventions that reduce differences in awareness and examine whether improving awareness and subsequent utilization of indicated genetic testing translates to improvements in health outcomes.

## Abbreviations

DTC: direct-to-consumer
HINTS: Health Information National Trends Survey
